# Monitoring of sandflies (Diptera: Psychodidae) and pathogen screening in Slovenia with habitat suitability modeling

**DOI:** 10.3389/fvets.2025.1603358

**Published:** 2025-07-25

**Authors:** Vladimir Ivović, Peter Glasnović, Sara Zupan, Tea Knapič, Tomi Trilar, Miša Korva, Nataša Knap, Urška Glinšek Biškup, Tatjana Avšič-Županc, Katja Adam

**Affiliations:** ^1^Faculty of Mathematics, Natural Sciences and Information Technologies, University of Primorska, Koper, Slovenia; ^2^Slovenian Museum of Natural History, Ljubljana, Slovenia; ^3^Institute of Microbiology and Immunology, Faculty of Medicine, University of Ljubljana, Ljubljana, Slovenia

**Keywords:** sandflies, monitoring, distribution, modeling, Slovenia

## Abstract

Sandflies (Diptera: Psychodidae: Phlebotominae) are important vectors of pathogens, including *Leishmania* parasites and phleboviruses, but their distribution and seasonal activity in Slovenia have not been sufficiently studied. This study presents a comprehensive three-year (2020–2022) surveillance programme aimed at assessing the diversity of sandfly species, their distribution, seasonal dynamics and potential role as vectors of pathogens. A total of 1,240 sandflies were collected at 43 sampling sites across Slovenia, identifying *Phlebotomus papatasi, P. neglectus, P. perniciosus* and *P. mascittii*. The highest abundance and species diversity were observed in the Mediterranean and Karst regions. Seasonal activity peaked in July, with population fluctuations influenced by climatic conditions. Molecular analyses for *Leishmania* parasites and phleboviruses showed no positive results, indicating a low prevalence of pathogens in the sampled populations. Predictive habitat models indicate that environmental factors, particularly temperature and precipitation, play a decisive role in the spread of sandflies. While *P. mascittii* has the largest ecological range, its vector competence remains uncertain. The results provide important insights into the ecology of sandflies in Slovenia and emphasize the need for continuous surveillance in the context of climate change and emerging vector-borne disease risks.

## Introduction

Sandflies are arthropods (Diptera: Psychodidae: Phlebotominae) of global importance as they are important vectors of several pathogens affecting humans and animals. These insects are known vectors of several *Leishmania* species that cause visceral and cutaneous leishmaniasis (1, 2), and of *Bartonella bacilliformis*, the causative agent of bartonellosis (3).

They are also vectors of phleboviruses such as Toscana virus (TOSV), sandfly fever Sicilian virus (SFSV), sandfly fever Naples virus (SFNV), and Cyprus virus (CYPV), which cause acute febrile or even central nervous system infections in humans (4–6).

Sandflies are delicate, hairy flies with long, slender legs that occur in a wide range of habitats, from sea level to altitudes of 2,800 m and from deserts, savannas, and open forests to dense tropical rainforests. These small, nocturnal insects require sugar as a source of energy, and only the female sandflies feed on blood as they need the nutrients to mature their eggs. The seasonal biting activity of European sandflies is mainly observed in summer (7). All sandflies breed in terrestrial habitats and are insects with relatively limited flight ability (8). In the last 10 years, several sandfly species have spread into European regions where they were not previously reported. This shift brings new public health challenges, as sandflies are vectors of *Leishmania* spp. and phleboviruses (9). Their expanding range is driven by a complex interplay of factors, including ongoing environmental and climate change, as well as increasing mobility of humans and animals. While climate change has always been a natural phenomenon, its rapid acceleration over the last century is unprecedented (10).

Six species of sandflies have been recorded in Slovenia and neighboring regions, which may influence the species composition and variability of local populations. These include *Phlebotomus* (*Transphlebotomus*) *mascittii* Grassi, 1908, *Phlebotomus* (*Larroussius*) *perniciosus* Newstead, 1911, *Phlebotomus* (*Larroussius*) *neglectus* Tonnoir, 1921, *Phlebotomus* (*Phlebotomus*) *papatasi* (Scopoli, 1786) and *Sergentomyia minuta* (Rondani, 1843).

*Phlebotomus mascittii* has been recorded in Slovenia (11) and in several neighboring countries, including Austria (12–14), Slovakia (15) and Croatia (16) as well as in parts of Germany and Belgium (17–21). *Phlebotomus perniciosus* is a common species in Italy and Croatia (22), while *P. neglectus* also occurs in neighboring Croatia (16, 23), Italy (24) and Hungary (25).

*Phlebotomus papatasi*, a predominantly endophilic and anthropophilic species, has a more southerly distribution in the region, with confirmed occurrences in Slovenia (11), Croatia (22) and southern Hungary (26).

Although *P. perfiliewi* has not yet been recorded in Slovenia, it has recently been reported in the Friuli-Venezia Giulia and Veneto regions in north-eastern Italy (27) and in Dalmatia, Croatia (22), indicating a possible expansion of the distribution area toward the Slovenian border.

Finally, *S. minuta* has been reported from Slovenia (11), Croatia (16), northern Italy (24) and Switzerland (28). However, as the females of this species mainly feed on reptiles, it is unlikely that they are involved in the transmission of pathogens to humans.

In Slovenia, research on sandflies as vectors of emerging pathogens has been relatively limited. Only a few studies have investigated their distribution, seasonal activity and possible role in the transmission of pathogens in selected areas. However, studies conducted in Slovenia and the former Yugoslavia have documented the presence of several medically important sandfly species, with the earliest records dating back to entomological surveys from the mid-20th century (29). More recent studies in Slovenia have documented the occurrence of *P. mascittii* and have highlighted illegal waste sites and peri-urban habitats as potential microfoci for sandfly proliferation (11, 30). In the broader region, human leishmaniasis has re-emerged as a public health concern, particularly in northern Italy, where retrospective analyses have identified hundreds of autochthonous cases over the past two decades (31). In Croatia, seroepidemiological surveys have demonstrated asymptomatic infections among residents in both endemic and non-endemic areas (32). Canine leishmaniasis remains widespread and of considerable veterinary relevance throughout the Balkans, with evidence of persistent transmission and a gradual northward spread in northern Italy, likely driven by environmental and climatic factors (33–37).

In addition to *Leishmania*, sandflies in Slovenia, Croatia, and Italy are competent vectors of several phleboviruses (38). Toscana virus, in particular, has emerged as an important cause of neuroinvasive disease, with multiple viral lineages detected in Croatia and endemic circulation documented across Italy (6, 39, 40). Earlier serological studies confirmed the widespread presence of sandfly fever viruses in the Adriatic region (41), and more recent data have demonstrated extensive Toscana virus exposure among humans and domestic animals (42).

Given Slovenia's diverse topography and climate, local climatic fluctuations could significantly influence sandfly populations and the pathogens they transmit. A standardized monitoring system is therefore essential to detect current trends and to predict future changes. The main objectives of this study were: (a) to document the sandfly species present in Slovenia; (b) to assess their current and potential distribution, potential range expansion, and abundance; and (c) to determine the presence or absence of viral and parasitic pathogens transmitted by sandflies. The results of this study will contribute to a clearer understanding of the epidemiological landscape of emerging pathogens in Slovenia and neighboring regions. In addition, these findings will support future research on the interactions between vectors, pathogens, and their environment, and will inform the development of improved surveillance and control strategies.

## Material and methods

### Study area

**Figure 1:**
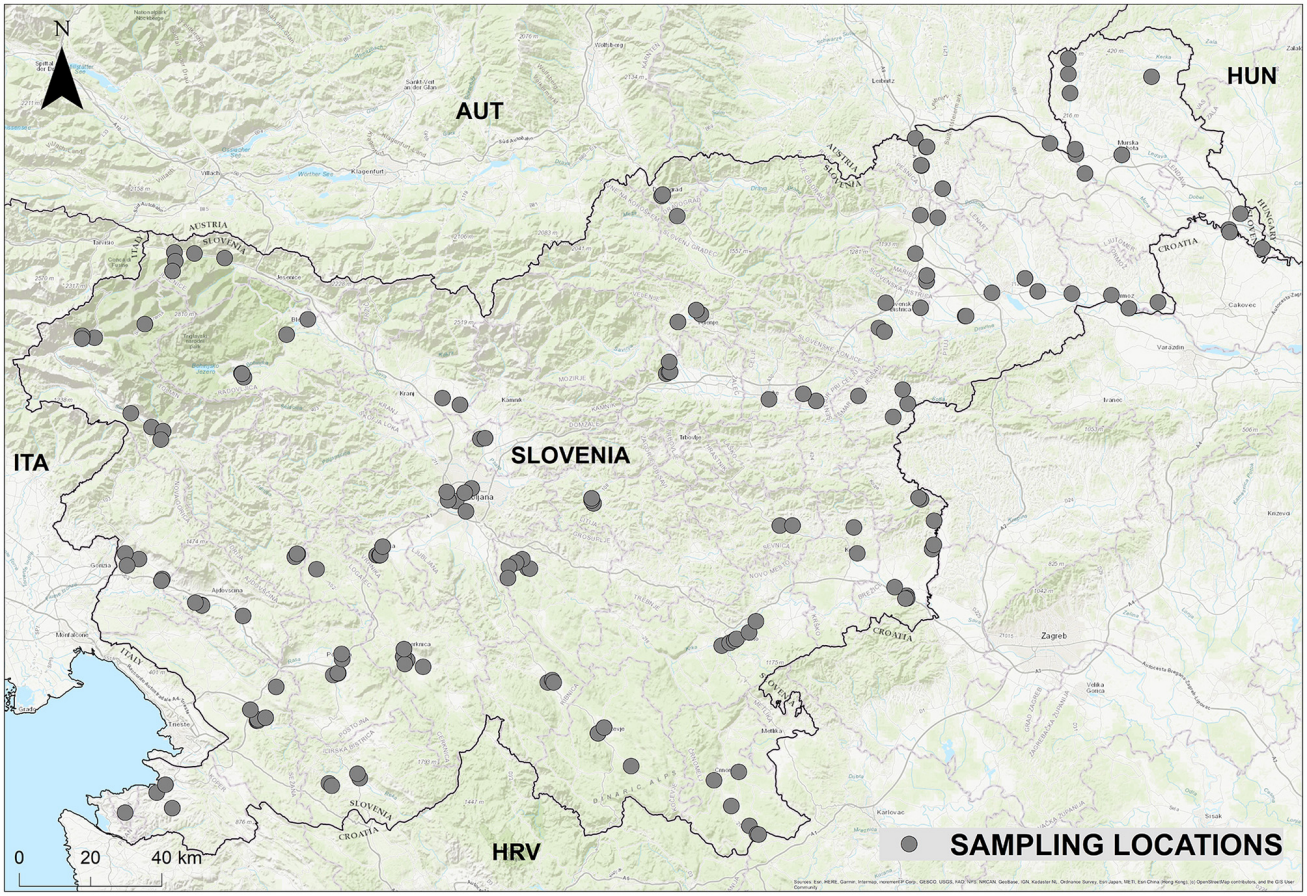
Sampling locations in Slovenia.

Prior to this study, data on the abundance of sandflies in Slovenia was incomplete, as these insects had only been studied in the coastal region and not in the continental part of the country until 2019. To fill this gap, a nationwide surveillance programme with systematic random sampling was carried out, covering a total of 226 sampling sites to ensure comprehensive geographical coverage (Figure 1). Sampling sites were selected to represent a variety of habitats, including suitable environments such as stone walls near human settlements, agricultural and tourist farms and animal enclosures, but also supposedly unsuitable habitats such as dense forests and areas near rivers or lakes. In this way, the presence or absence of sandflies could be detected in unexpected locations.

The site selection was based on the expectation of high species diversity. We prioritized locations that would maximize the detection of different sandfly species and their population densities, focusing on areas with historically high sandfly activity, such as the Slovenian Karst region (11, 29). In addition, we have strategically selected locations where larger numbers of sandflies have been detected in the past and which are close to regions in the neighboring countries of Italy and Croatia where leishmaniasis has occurred. This structured approach ensured a scientifically rigorous assessment of sandfly populations in Slovenia.

The study followed a longitudinal design, with repeated sampling across three sandfly seasons (2020–2022). To ensure broad spatial coverage and habitat diversity, sites were distributed across different geographic regions and environmental conditions. The temporal framework allowed for long-term monitoring of seasonal population dynamics. Systematic sampling provided reliable data on species presence, seasonal activity, and population trends. Detailed information on the individual sampling locality is available in the table in the Appendix.

### Trapping methods

Fieldwork was conducted annually from May 1st to October 31st between 2020 and 2022. Each month, two CDC miniature light traps (John W. Hock Company, Florida, USA) were set at each sampling site. In addition to these traps, we also used BG-Sentinel traps (Biogents, USA) and CDC gravid traps (John W. Hock Company, Florida, USA) as part of a broader entomological surveillance programme to monitor mosquito populations at the same locations. While CDC miniature light traps were the primary method for collecting adult sandflies, we occasionally captured them in both BG-Sentinel traps and CDC gravid traps for mosquitoes.

Sandflies require a certain amount of moisture to maintain their life cycle and are most often found near hosts from which they can take a blood meal. Therefore, we mainly placed the traps near or inside buildings where domestic animals, such as chickens, rabbits and dogs were kept. We also placed the traps near dry stone walls, which are ideal resting places for sandflies in Mediterranean environments.

To better understand the spatial distribution of sandflies, we also set traps in areas typically not associated with their presence. This approach was crucial for the development of models to predict possible changes in their distribution in Slovenia. We did not attempt to sample sandfly larvae as it is difficult to find them in the wild. They usually hide in organic material outdoors or inside barns, chicken coops or rabbit hutches (2).

### Identification of sandflies based on morphology

Sandflies were identified based on morphology using identification keys (43–45). The identification of the species of adult sandflies requires the examination of internal structures, such as the arrangement of the teeth on the cibarial (pharyngeal) armature, the shape of the spermathecae in females, and the structure of the external genitalia (terminalia) in males.

As one of the aims of our study was to analyse the captured sandflies for the presence of *Leishmania* parasites and phleboviruses, all specimens were immediately placed in a freezer upon return from the field and stored at −80°C until dissection. Dissections were performed as quickly as possible on ice plates to preserve viral RNA. The head and several posterior segments of the abdomen were carefully separated from each fly using fine entomological or insulin needles on a microscope slide and then preserved for the preparation of semi-permanent slides, which were later used for morphological identification. The transparency of the chitinous parts was achieved by heating in the Marc-André solution. The rest of the body was pooled according to location, species and sex and stored at −80°C for molecular analysis.

Before identification, the head and posterior segments of each specimen were immersed in a drop of low-viscosity CMP-9 medium (Polyscience, Cat. No. 16299). The head was mounted dorsally, the male genitalia laterally and the spermathecae anteriorly. For easier identification, the spermathecae of some females were individually prepared directly in the Marc-André solution.

### Habitat suitability modeling

To create maps of the potential distribution of sandflies, we used data on their presence at specific locations across all three sampling years. To reduce bias caused by the uneven distribution of sampling locations, we performed spatial filtering as suggested by (46), counting multiple records within a 1 km radius as a single occurrence point. The data were filtered using the “ecospat” package (47).

For the input data in the modeling process, we used 19 bioclimatic variables from the CHELSA climate database (48) (https://chelsa-climate.org/). We selected the CHELSA bioclimate variables because they have been shown to be a better choice compared to the more commonly used WorldClim variables in a previous study on mosquito modeling in Slovenia (49). Additionally, we incorporated an elevation layer from the WorldClim website (www.worldclim.org) and a CORINE land cover layer from the European Environment Agency website (https://www.eea.europa.eu/help/faq/what-is-corine-land-cover) to develop the model. The spatial resolution of the environmental variable layers was 30 arcseconds, corresponding to ~1 × 1 km. We checked predictors for collinearity to remove highly correlated and thus redundant environmental variables. To achieve this, we calculated the variance inflation factor (VIF ≥ 5) and *post-hoc* Pearson correlation coefficient between variables. VIF analysis was performed using the R package “usdm” (50), while Pearson coefficients were calculated and visualized using the R package “GGally” (51).

Due to the limited number of occurrences, we decided to create models using the MAXENT algorithm (52), which has been shown to provide better results with smaller amounts of occurrence data (53, 54).

We considered the approach proposed by (55) for modeling rare species and created several small bivariate models and their ensemble as the final model. The modeling procedure was performed using the R package “ecospat” (47). All models were evaluated based on the Boyce index. Models were based on the ensemble prediction procedure. Models with highest values of Boyce index were considered for the final model. For the visualization of final maps and for spatial procedures we used the spatial tools of ESRI ArcGIS, ver.10.7.

### Molecular detection of *Leishmania* parasites and phleboviruses

The dissected specimens were pooled together based on collection site, date, species and sex. The pooled material consisted of the thorax and abdomen sections of dissected individuals of the same species and sex. In contrast, the non-dissected specimens were pooled only by locality, date and sex and stored at −80°C until processing. As the primary aim of this study was the detection of phleboviruses, we dissected every second specimen to obtain internal tissues with potentially higher viral RNA concentrations for reliable molecular analysis. Each pool of sandflies was homogenized in 600 μL of RPMI medium using a Tissue Lyser (Retsch for Qiagen, Hilden, Germany). Nucleic acid was extracted from 200 μL of the sandfly homogenate using the BioRobot EZ1-XL Advanced (Qiagen, Germany) and the EZ1 Virus Mini Kit v2.0 (Qiagen, Germany) and eluted in 60 μL. The residual homogenate was stored for further analysis. We did not check RNA integrity after extraction, as all samples were transported on dry ice and immediately stored at −80°C, and extractions were performed within 2 weeks. However, internal control amplification in the PCR assays confirmed the presence of amplifiable RNA in all analyzed pools.

*Leishmania* DNA was detected by real-time PCR using the Primerdesign™ *Leishmania* Genesig^®^ Advanced Kit (Genesig, Primerdesign, UK) targeting Cytochrome b (cytb) kinetoplast. Reactions were performed in a total volume of 20 μL containing 5 μL DNA, 5 μL TaqMan^®^ Fast Virus 1-Step Master Mix (Applied Biosystems, Thermo Fisher Scientific, Grand Island, NY, USA), 1 μL *Leishmania* primer/probe mix on the StepOne (Plus) Real-Time PCR System (Applied Biosystems, Thermo Fisher Scientific, USA). Cycling conditions were as follows: 50°C for 5 min, 95°C for 20 s, 50 cycles of 95°C for 3 s and 60°C for 30 s.

The sandfly pools were analyzed using Toscana virus-specific real-time RT-PCR as described by (56). Real-time RT-PCR was performed using a QuantStudio 7 system (Applied Biosystems, USA). Reactions were performed in a total volume of 20 μl and contained 5 μl of RNA, 2.5 μl of TaqMan^®^ Fast Virus 1-Step Master Mix (Applied Biosystems, Thermo Fisher Scientific, Grand Island, NY, USA), 0.5 μmol of each primer and 0.3 μmol of probe, and water. Cycling conditions were as follows: 50°C for 5 min, 95°C for 20 s, followed by 40 cycles of 95°C for 3 s and 60°C for 30 s.

In addition, the sandfly pools were analyzed with a RT-PCR developed (57) using Phlebo forward primers 1 and 2 and Phlebo reverse primers, which allowed the amplification of a 370 bp PCR product of the S segment of viruses of the genus Phlebovirus (57). The reactions were performed in a total volume of 20 μL and contained 5 μL RNA, 10 μL PrimeScrtipt™ One Step RT-PCR Kit Buffer and 1 μL PrimeScript 1 Step Enzyme Mix (TaKaRa) and 1 μM of each primer. Cycling conditions were as follows: 50°C for 30 min, 94°C for 2 min, 55 cycles of 94°C for 30 s, 55°C for 30 s and 72°C for 30 s.

## Results

During the study period, a total of 1,240 individuals were caught at 43 of 226 sampling sites. In 2020, we caught 552 sandflies, followed by 240 individuals in 2021 and 448 individuals in 2022. 670 individuals were identified to a species level based on morphology. The rest were pooled according to sex and location and prepared for molecular analysis to identify pathogenic microorganisms.

Based on morphological characteristics, we identified four species of sandflies, *Phlebotomus papatasi, P. neglectus, P. perniciosus* and *P. mascitii*.

**Figure 2:**
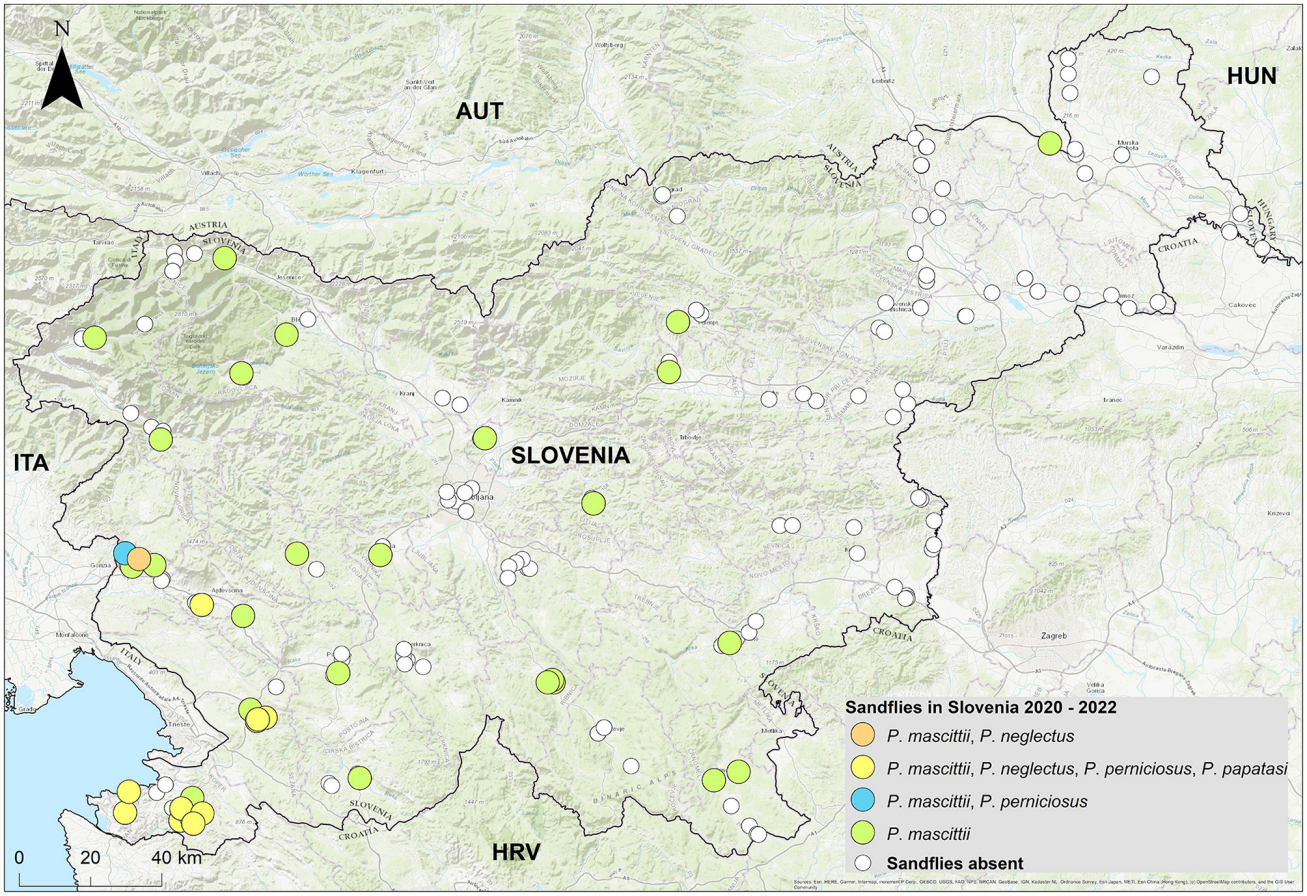
Distribution and population structure of sandflies in Slovenia.

**Figure 3:**
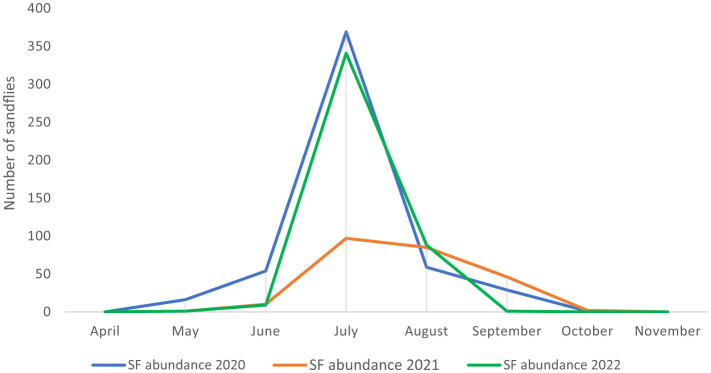
Seasonal activity of sandflies in Slovenia from 2020 to 2022.

Phlebotomine sandflies were found throughout the country (Figure 2), with notable differences in population structure and spatial distribution. *Phlebotomus neglectus, P. perniciosus* and *P. papatasi* were predominantly found in the Mediterranean and Karst regions of Slovenian Istria, while *P. mascitii* was found in all surveyed locations. *Phlebotomus mascittii*, a generally not abundant species, was found most frequently in Slovenian Istria, with significant records from the Medljan and Velike Žablje areas (Appendix 1). Data from 3 years of research show that sandfly activity peaks in July (Figure 3). As not all specimens could be identified to species level, a more precise seasonal analysis of species activity is not conclusive.

### Prediction maps

**Figure 4:**
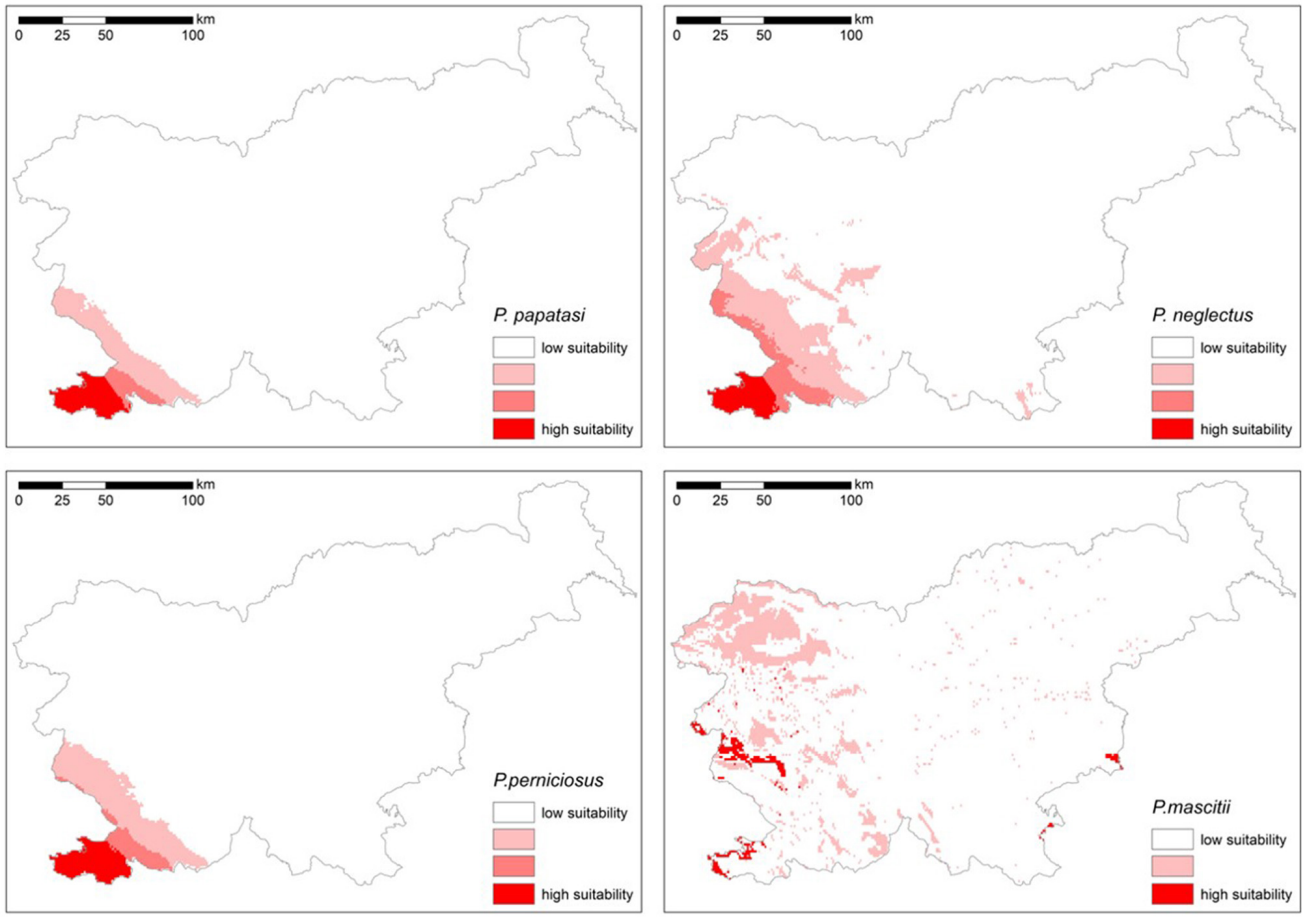
Suitability of areas in Slovenia for the settlement of *P. papatasi, P. neglectus, P. perniciosus and P. mascitii* (suitability increases from white color (inadequate) to dark red color (very suitable).

The models were created considering the following predictor variables, which showed low collinearity: elevation, Corine land cover, isothermality (bio3), maximum temperature of the warmest month (bio5), precipitation seasonality (bio15), precipitation of the wettest quarter (bio16) and precipitation of the warmest quarter (bio18) (Figure 4).

The models for *P. perniciosus* and *P. neglectus* were created using an ensemble of bivariate models that met a Boyce index threshold of 0.5 and 0.4, respectively. Despite the optimization efforts, a certain degree of model overfitting remained in the results. For *P. papatasi* and *P. mascittii*, which have a similar distribution to the first two species, the ensemble was based on bivariate models with a higher Boyce index threshold of 0.6 and 0.7, respectively.

The maps show the suitability of different regions in Slovenia for the potential distribution of sandfly species (Figure 4). The most favorable environmental conditions for all four species are found in the south-western part of the country, particularly in low-lying areas and along the coast. While three of the species are largely restricted to the sub-Mediterranean region, *P. mascittii* seems to have a larger distribution potential, extending to ecologically favorable areas in the continental part of Slovenia, especially in the warmer lowlands in the east. In contrast, the central, eastern and northern regions, which are represented by white areas on the maps, are less suitable (Figure 4). This indicates that the environmental conditions in these regions are less favorable for the establishment of sandfly populations.

### Molecular analysis

A total of 1,217 sandfly specimens were processed for molecular analysis and organized into 179 pools. Twenty-three additional samples were neither suitable for morphological identification nor for molecular testing. No pathogens were detected in any of the samples analyzed, including 551 specimens (94 pools) from 2020, 218 specimens (35 pools) from 2021 and 448 specimens (50 pools) from 2022.

## Discussion

Sandflies, important vectors of pathogens that cause diseases such as leishmaniasis and phleboviruses, are changing their distribution in Europe due to climate change, urbanization and changes in land use (9, 58). These factors create more favorable conditions for the survival and reproduction of sandflies (59). As their distribution expands, the risk of leishmaniasis outbreaks in newly colonized regions increases.

Important vector species such as *P. perniciosus* and *P. neglectus*, which were previously restricted to Mediterranean areas, have been detected further north, including in Germany and Switzerland (19, 60). *Phlebotomus mascitii*, whose vector potential is still unconfirmed, is also spreading north. Although its abundance is generally low, this species is found as far as Switzerland and Slovakia (15, 60). Its spread is probably due to milder winters and longer warm seasons, which have transformed previously unsuitable areas into viable habitats (22).

In Slovenia, sandflies are widespread throughout the country, although there are notable regional differences in population structure. *Phlebotomus neglectus, P. perniciosus* and *P. papatasi* are mainly found in the Mediterranean and Karst regions of Slovenian Istria, while *P. mascitii* is present in all areas studied. However, the greatest species diversity is observed in areas with a Mediterranean climate. The data from 3 years of research show that sandfly activity peaks in July (Figure 3), similar to what is observed in the neighboring Italian regions of Friuli-Venezia Giulia and Veneto (27) and in Austria (12). Our results suggest that the sandflies in this region only produce one generation per season due to the climatic conditions.

According to meteorological data from the Slovenian Environment Agency, the average annual temperature in Slovenia was 14.37°C in 2020, 13.85°C in 2021 and 14.86°C in 2022. The temperature deviation for 2021 was −0.7°C compared to the long-term average. Exceptionally high precipitation was recorded in 2020, followed by lower winter temperatures in 2020/2021 (61). These climatic conditions may have influenced the population dynamics of phlebotomine sandflies, especially in Slovenian Istria and the Karst region, where they are most abundant. Increased precipitation and lower winter temperatures probably affected the suitability of breeding sites and larval survival rates, which may have contributed to the changes in population density in 2021. A similar negative correlation between precipitation and abundance of *P. perniciosus* was observed in the Murcia region of Spain and north-eastern Italy (62, 63). As a result, the number of sandflies caught in 2021 was significantly lower than in 2020 and 2022, highlighting the impact of unfavorable climatic conditions on their population dynamics.

Environment suitability models show that the studied species have different habitat preferences. *Phlebotomus papatasi, P. neglectus* and *P. perniciosus* show a strong preference for the south-western part of Slovenia, particularly in the coastal and Karst regions, and are very limited for other areas. In contrast, *P. mascitii* has a much broader potential range, covering almost the entire country. This suggests that *P. mascitii* can thrive in a range of environmental conditions, including higher altitudes and potentially cooler or wetter regions. These distribution patterns are consistent with the environmental preferences observed in Austria and Slovakia (12, 15). Despite its wide distribution range, *P. mascitii* is not very abundant and is most frequently detected in Slovenian Istria, with significant populations found in Medljan and Velike Žablje.

The observed ecological differences between sandfly species in Slovenia may influence their role as disease vectors and their impact on public health. *P. papatasi, P. neglectus* and *P. perniciosus* are adapted to warm, dry regions with Mediterranean climate characteristics, while *P. mascitii* shows greater ecological flexibility. In the Mediterranean regions, *P. mascitii* is thought to be a cave-dwelling species (20), while in Central Europe it is often found in barns, chicken coops and sheds close to human dwellings and animals (12). At sites in Slovenia, this species was most frequently found in traps placed in the immediate vicinity of chicken coops or rabbit cages. *P. mascitii* is thought to be autogenous (64), although its biology remains poorly understood due to difficulties in laboratory maintenance.

Although *P. mascitii* is found near domestic animal shelters and burrows of small rodents, its low natural density is unlikely to sustain the transmission cycle of *Leishmania* infantum alone. Nevertheless, it may play a localized role in the transmission of *L. infantum*, particularly in cases of imported canine leishmaniasis. Its presence in suburban areas and animal enclosures indicates a possible epidemiological importance in regions where competent vectors are scarce.

As far as leishmaniasis in humans is concerned, no autochthonous cases have been reported to date, only imported cases from endemic Mediterranean areas (65). There is evidence that cases of canine leishmaniasis have occurred in Slovenia in the last 5 years (66, 67), similar to the northern regions of neighboring Italy and Hungary (68, 69). Unlike in Italy and Croatia, however, we were unable to detect any *Leishmania* parasites or phleboviruses in the sandfly samples we analyzed (38, 70).

The results of this three-year surveillance show the ecological differences between sandfly species in Slovenia and their potential impact on disease transmission. Understanding these differences is essential for predicting future vector dynamics and developing targeted control measures to reduce disease risks.

## Conclusion

This study provides a comprehensive assessment of the distribution of sandflies in Slovenia and shows clear regional differences in the abundance of the species and their habitat preferences. The results confirm that climatic and environmental conditions significantly influence sandfly populations, with *P. mascitii* having the largest ecological range. While the vector competence of this species remains uncertain, its presence near animal enclosures suggests a possible epidemiological relevance. The absence of *Leishmania* and phleboviruses in the samples is a reassuring result. However, because of the constant expansion of the distribution area of the known vector species, continuous monitoring is essential. Future research should focus on the potential role of *P. mascitii* in pathogen transmission and the impact of climate change on vector dynamics. Effective surveillance and proactive control measures will be crucial to mitigate the risk of emerging sandfly-borne diseases not only in Slovenia but throughout the Mediterranean region.

## Data Availability

The raw data supporting the conclusions of this article will be made available by the authors, without undue reservation.

## References

[1] Berriatua E Maia C Conceição C Özbel Y Töz S Baneth G. Leishmaniases in the European Union and neighboring countries. Emerg Infect Dis. (2021) 27:1723–7. 10.3201/eid2706.210239 34013857 PMC8153892

[2] Killick-Kendrick R. The biology and control of phlebotomine sandflies. Clin Dermatol. (1999) 17:279–89. 10384867 10.1016/s0738-081x(99)00046-2

[3] Billeter SA Levy MG Chomel BB Breitschwerdt EB. Vector transmission of *Bartonella* species with emphasis on the potential for tick transmission. Med Vet Entomol. (2008) 22:1–15. 10.1111/j.1365-2915.2008.00713.x 18380649

[4] Depaquit J Grandadam M Fouque F Andry P Peyrefitte C. Arthropod-borne viruses transmitted by phlebotomine sand flies in Europe: a review. Euro Surveill. (2010) 15:20782. 10.2807/ese.15.10.19507-en 20403307

[5] Papa A Konstantinou G Pavlidou V Antoniadis A. Sandfly fever virus outbreak in Cyprus. Clin Microbiol Infect. (2006) 12:192–4. 10.1111/j.1469-0691.2005.01330.x 16441462

[6] Charrel RN Gallian P Navarro-Mari JM Nicoletti L Papa A Sánchez-Seco MP. Emergence of Toscana virus in Europe. Emerg Infect Dis. (2005) 11:1657–63. 10.3201/eid1111.050869 16318715 PMC3367371

[7] Ready PD. Leishmaniasis emergence and climate change. Rev Sci Tech. (2008) 27:399–412. 10.20506/rst.27.2.1803 18819668

[8] Lane RP. Sandflies (Phlebotominae). In:Lane RP Crosskey RW, editors. Medical Insects and Arachnids. Dordrecht: Springer Netherlands (1993). p. 78–119.

[9] Braks M Schaffner F Medlock JM Berriatua E Balenghien T Mihalca AD. VectorNet: putting vectors on the map. Front Public Health. (2022) 10:809763. 10.3389/fpubh.2022.809763 35444989 PMC9013813

[10] World Meteorological Organisation (WMO). (2024). Available online at: https://wmo.int/news/media-centre/2024-track-be-hottest-year-record-warming-temporarily-hits-15degc (Accessed March 15, 2025).

[11] Ivović V Adam K Zupan S BuŽan E. Illegal waste sites as a potential micro foci of Mediterranean leishmaniasis: first records of Phlebotomine sand flies (Diptera: Psychodidae) from Slovenia. Acta Vet. (2015) 65:348–57. 10.1515/acve-2015-0029

[12] Kniha E Dvorák V Halada P Milchram M Obwaller AG Kuhls K. Integrative approach to *Phlebotomus mascittii* Grassi, 1908: First record in Vienna with new morphological and molecular insights. Pathogens. (2020) 9:1032. 10.3390/pathogens9121032 33317097 PMC7764109

[13] Kniha E Milchram M Dvorák V Halada P Obwaller AG Poeppl W. Ecology, seasonality and host preferences of Austrian *Phlebotomus* (*Transphlebotomus*) *mascittii* Grassi, 1908, populations. Parasites Vectors. (2021) 14:291. 10.1186/s13071-021-04787-2 34051839 PMC8164323

[14] Obwaller AG Karakus M Poeppl W Töz S Özbel Y Aspöck H. Could *Phlebotomus mascittii* play a role as a natural vector for Leishmania infantum? New data. Parasites Vect. (2016) 9:1–6. 27542911 10.1186/s13071-016-1750-8 PMC4992248

[15] Dvorak V Hlavackova K Kocisova A Volf P. First record of *Phlebotomus* (*Transphlebotomus*) *mascittii* in Slovakia. Parasite. (2016) 23:48. 10.1051/parasite/2016050 27849514 PMC5112768

[16] Bosnić S Gradoni L Khoury C Maroli MA. Review of leishmaniasis in Dalmatia (Croatia) and results from recent surveys on phlebotomine sand flies in three southern counties. Acta Trop. (2006) 99:42–9. 10.1016/j.actatropica.2006.06.009 16876101

[17] Oerther S Jöst H Heitmann A Lühken R Krüger A Steinhausen I. Phlebotomine sand flies in Southwest Germany: an update with records in new locations. Parasites Vectors. (2020) 13:1–8. 10.1186/s13071-020-04058-6 32312300 PMC7171781

[18] Melaun C Krüger A Werblow A Klimpel S. New record of the suspected leishmaniasis vector *Phlebotomus* (*Transphlebotomus*) *mascittii* Grassi, 1908 (Diptera: Psychodidae: Phlebotominae) – the northernmost phlebotomine sandfly occurrence in the Palearctic region. Parasitol Res. (2014) 113:2295–301. 10.1007/s00436-014-3884-y 24737399

[19] Naucke T Menn B Massberg D Lorentz S. Sandflies and leishmaniasis in Germany. Parasitol Res. (2008) 103:65–8. 10.1007/s00436-008-1052-y 19030887

[20] Depaquit J Naucke TJ Schmitt C Ferté H Léger N A. molecular analysis of the subgenus *Transphlebotomus* Artemiev, 1984 (Phlebotomus, Diptera, Psychodidae) inferred from ND4 mtDNA with new northern records of *Phlebotomus mascittii* Grassi, 1908. Parasitol Res. (2005) 95:113–6. 10.1007/s00436-004-1254-x 15666186

[21] Naucke T Pesson B. Presence of *Phlebotomus* (*Transphlebotomus*) *mascittii* Grassi, 1908 (Diptera: Psychodidae) in Germany. Parasitol Res. (2000) 86:335–6. 10.1007/s004360050053 10780745

[22] Dvorak V Kasap OE Ivovic V Mikov O Stefanovska J Martinkovic F. Sand flies (Diptera: Psychodidae) in eight Balkan countries: historical review and region-wide entomological survey. Parasites Vectors. (2020) 13:573. 10.1186/s13071-020-04396-6 33176888 PMC7661266

[23] Biševac L Miščević Z Milutinović M A. contribution to the investigations of sand fly fauna (Diptera, Phlebotomidae) of the island of Mljet, Croatia, Yugoslavia. Acta Vet (Belgrade). (1990) 40:49–54.

[24] Maroli M Feliciangeli MD Bichaud L Charrel RN Grandoni L. Phlebotomine sandflies and the spreading of leishmaniases and other diseases of public health concern. Med Vet Entomol. (2013) 27:123–47. 10.1111/j.1365-2915.2012.01034.x 22924419

[25] Farkas R Tánczos B Bongiorno G Maroli M Dereure J Ready PD. First surveys to investigate the presence of canine leishmaniasis and its phlebotomine vectors in Hungary. Vector Borne Zoonotic Dis. (2011) 11:823–34. 10.1089/vbz.2010.0216 21254904

[26] Trájer AJ. Checklist, distribution maps, bibliography of the Hungarian Phlebotomus (Diptera: Psychodidae) fauna complementing with the climate profile of the recent sandfly distribution areas in Hungary. Folia faunistica Slovaca. (2017) 22:7–12.

[27] Michelutti A Toniolo F Bertola M Grillini M Simonato G Ravagnan S. Occurrence of phlebotomine sand flies (Diptera: Psychodidae) in the northeastern plain of Italy. Parasit Vectors. (2021) 14:164. 10.1186/s13071-021-04652-2 33761950 PMC7992963

[28] Knechtli R Jenni L. Distribution and relative density of three sandfly (Diptera: Phlebotominae) species in southern Switzerland. Ann Parasitol Hum Comp. (1989) 64:53–63.

[29] Simić C. Contribution à la connaissance de Phlébotomes en Yougoslavie. Glas Srp Akad Nauka Med. (1951) 4:17–34.

[30] Praprotnik E Zupan S Ivović V. Morphological and molecular identification of *Phlebotomus mascittii* Grassi, 1908 populations from Slovenia. J Med Entomol. (2019) 56:565–8. 10.1093/jme/tjy176 30289462

[31] Todeschini R Musti MA Pandolfi P Troncatti M Baldini M Resi D. Re-emergence of human leishmaniasis in northern Italy, 2004 to 2022: a retrospective analysis. Euro Surveill. (2024) 29:2300190. 10.2807/1560-7917.ES.2024.29.4.2300190 38275016 PMC10986649

[32] Šiško-Kraljević K Jerončić A Mohar B Punda-Polić V. Asymptomatic *Leishmania infantum* infections in humans living in endemic and non-endemic areas of Croatia, 2007 to 2009. Euro Surveill. (2013) 18:20533. 10.2807/1560-7917.ES2013.18.28.20533 23929119

[33] Taddei R Bregoli A Galletti G Carra E Fiorentini L Fontana MC. Wildlife hosts of *Leishmania infantum* in a re-emerging focus of human leishmaniasis, in Emilia-Romagna, Northeast Italy. Pathogens. (2022) 11:1308. 10.3390/pathogens11111308 36365059 PMC9697138

[34] Vaselek S. Canine leishmaniasis in Balkan – a review of occurrence and epidemiology. Acta Trop. (2021) 224:106110. 10.1016/j.actatropica.2021.106110 34450057

[35] Morosetti G Toson M Trevisiol K Idrizi I Natale A Lucchese L. Canine leishmaniosis in the Italian northeastern Alps: a survey to assess serological prevalence in dogs and distribution of phlebotomine sand flies in the Autonomous Province of Bolzano–South Tyrol, Italy. Vet Parasitol Reg Stud Reports. (2020) 21:100432. 10.1016/j.vprsr.2020.100432 32862903

[36] Živičnjak T Martinković F Khoury C Bongiorno G Bosnić S Lukačević D. Serological and entomological studies of canine leishmaniosis in Croatia. Vet Arhiv. (2011) 81:99–110.

[37] Živičnjak T Martinković F Marinculić A Mrljak V Kucer N Matijatko V. seroepidemiologic survey of canine visceral leishmaniosis among apparently healthy dogs in Croatia. Vet Parasitol. (2005) 131:35–43. 10.1016/j.vetpar.2005.04.036 15946800

[38] Ayhan N Charrel RN. Emergent sand fly–borne phleboviruses in the Balkan region. Emerg Infect Dis. (2018) 24:2324–30. 10.3201/eid2412.171626

[39] Ayhan N Alten B Ivovic V Cvetkovikj A Stefanovska J Martinkovic F. Field surveys in Croatia and North Macedonia reveal two novel phleboviruses circulating in sandflies. J Gen Virol. (2021) 102:001674. 10.1099/jgv.0.001674 34797756

[40] Ayhan N Alten B Ivovic V Martinkovic F Kasap OE Ozbel Y. Cocirculation of Two Lineages of Toscana Virus in Croatia. Front Public Health. (2017) 5:336. 10.3389/fpubh.2017.00336 29312917 PMC5732939

[41] Tesh RB Saidi S Gajdamovič SJ Rodhain F Vesenjak-Hirjan JELKA. Serological studies of the epidemiology of sandfly fever in the Old World. Bull World Health Organ. (1976) 54:663. 829416 PMC2366583

[42] European Centre for Disease Prevention and Control (ECDC). Toscana Virus Infection. (2023). Available online at: https://www.ecdc.europa.eu/en/toscana-virus-infection (Accessed July 1, 2025).

[43] Artemiev MM Neronov VM. Distribution and ecology of sandflies of the Old World (genus Phlebotomus). Moscow: Institute of Evolutionary Morphology and Animal Ecology, USSR Academy of Sciences (1984). p. 207.

[44] Lewis D Lane R. A taxonomic review of *Phlebotomus* (*Idiophlebotomus*) (Psychodidae). Syst Entomol. (1976) 1:53–60.

[45] Perfiliew PP. Fauna of the USSR Diptera, Phlebotomidae. Fauna of the USSR, Vol. 3, No. 2. Moskow: Academy of Science USSR, Institute for Zoology, Science (1966). In Russian.

[46] Kramer-Schadt S Niedballa J Pilgrim JD Schröder B Lindenborn J Reinfelder V. The importance of correcting for sampling bias in MaxEnt species distribution models. Diversity Distrib. (2013) 19:1366–79. 10.1111/ddi.12096

[47] Di Cola V Broennimann O Petitpierre B Breiner FT D'Amen M Randin C. ecospat: an R package to support spatial analyses and modeling of species niches and distributions. Ecography. (2017) 40:774–87. 10.1111/ecog.02671

[48] Karger DN Conrad O Böhner J Kawohl T Kreft H Soria-Auza RW. Climatologies at high resolution for the earth's land surface areas. Sci Data. (2017) 4:170122. 10.1038/sdata.2017.122 28872642 PMC5584396

[49] Kalan K Ivović V Glasnović P Buzan E. Presence and potential distribution of *Aedes albopictus* and *Aedes japonicus japonicus* (Diptera: Culicidae) in Slovenia. J Med Entomol. (2017) 54:1510–8. 28968852 10.1093/jme/tjx150

[50] Naimi B Hamm NAS Groen TA Skidmore AK Toxopeus AG. Where is positional uncertainty a problem for species distribution modelling? Ecography. (2014) 37:191–203. 10.1111/j.1600-0587.2013.00205.x

[51] Wickham H. ggplot2: Elegant Graphics for Data Analysis. New York: Springer-Verlag (2016).

[52] Phillips JS Anderson RP Schapire RE. Maximum entropy modelling of species geographic distributions. Ecol Model. (2006) 190:231–59. 10.1016/j.ecolmodel.2005.03.026

[53] Rinnhofer LJ Roura-Pascual N Arthofer W Dejaco T Thaler-Knoflach B Wachter GA. Iterative species distribution modelling and ground validation in endemism research: an Alpine jumping bristletail example. Biodivers Conserv. (2012) 21:2845–63. 10.1007/s10531-012-0341-z

[54] Pearson RG Raxworthy CJ Nakamura M Peterson AT. Predicting species distributions from small numbers of occurrence records: a test case using cryptic geckos in Madagascar. J Biogeogr. (2007) 34:102–17. 10.1111/j.1365-2699.2006.01594.x

[55] Breiner FT Guisan A Bergamini A Nobis MP. Overcoming limitations of modelling rare species by using ensembles of small models. Methods Ecol Evol. (2015) 6:1210–8. 10.1111/2041-210X.12403

[56] Pérez-Ruiz M Collao X Navarro-Marí JM Tenorio A. Reverse transcription, real-time PCR assay for detection of Toscana virus. J Clin Virol. (2007) 39:276–81. 10.1016/j.jcv.2007.05.003 17584525

[57] Lambert AJ Lanciotti RS. Consensus amplification and novel multiplex sequencing method for S segment species identification of 47 viruses of the *Orthobunyavirus, Phlebovirus*, and *Nairovirus* genera of the family Bunyaviridae. J Clin Microbiol. (2009) 47:2398–404. 10.1128/JCM.00182-09 19535518 PMC2725646

[58] Medlock JM Hansford KM Van Bortel W Zeller H Alten B A. summary of the evidence for the change in European distribution of phlebotomine sand flies (Diptera: Psychodidae) of public health importance. J Vector Ecol. (2014) 39:72–7. 10.1111/j.1948-7134.2014.12072.x 24820558

[59] Alten B Maia C Afonso MO Campino L Jimenez M Gonzalez E. Seasonal dynamics of phlebotomine sand fly species proven vectors of Mediterranean leishmaniasis caused by Leishmania infantum. PLoS Negl Trop Dis. (2016) 10:e0004458. 10.1371/journal.pntd.0004458 26900688 PMC4762948

[60] Schaffner F Silaghi C Verhulst NO Depaquit J Mathis A. The Phlebotomine sand fly fauna of Switzerland revisited. Med Vet Entomol. (2024) 38:13–22. 10.1111/mve.12690 37642138

[61] Slovenian Environment Agency (ARSO). Ministry of the Environment, Climate and Energy. Ljubljana: Slovenian Environmental Agency (ARSO) (2025). Available online at: https://meteo.arso.gov.si/met/sl/archive/ (Accessed March 15, 2025).

[62] Risueño J Muñoz C Pérez-Cutillas P Goyena E Gonzalvez M Ortuno M. Understanding *Phlebotomus perniciosus* abundance in south-east Spain: assessing the role of environmental and anthropic factors. Parasites Vectors. (2017) 10:189. 10.1186/s13071-017-2135-3 28420407 PMC5395901

[63] Signorini M Cassini R Drigo M. Frangipane di Regalbono A, Pietrobelli M, Montarsi F, Stensgaard AS. Ecological niche model of Phlebotomus perniciosus, the main vector of canine leishmaniasis in north-eastern Italy. Geospat Health. (2014) 9:193–201. 10.4081/gh.2014.16 25545936

[64] Ready PD Ready PA. Prevalence of *Phlebotomus* spp. in southern France: sampling bias due to different man-biting habits and autogeny. Ann Trop Med Parasitol. (1981) 75:475–6. 7305516 10.1080/00034983.1981.11687471

[65] National Institute of Public Health (NIJZ). Monitoring of Infectious Diseases. Reports from 2015–2017 (2017). Available online at: https://nijz.si/nalezljive-bolezni/epidemiolosko-spremljanje-nalezljivih-bolezni-letna-in-cetrtletna-porocila-arhiv/

[66] Kotnik T Ivović V. Living on the edge: Border countries should have strict veterinary and health policy on leishmaniasis. In:Claborn D, editor. The Epidemiology and Ecology of Leishmaniasis. London: InTech (2017). p. 3–16.

[67] Kotnik T Moreno J Šoba B Krt B Skvarč M Vergles Rataj A. Canine leishmaniasis prevalence in the Slovenian dog population. J Vet Res. (2021) 65:161–7. 10.2478/jvetres-2021-0028 34250300 PMC8256472

[68] Mendoza-Roldan J Benelli G Panarese R Iatta R Furlanello T Beugnet F. Leishmania infantum and Dirofilaria immitis infections in Italy, 2009–2019: changing distribution patterns. Parasites Vectors. (2020) 13:193. 10.1186/s13071-020-04063-9 32293524 PMC7161282

[69] Tánczos B Balogh N Király L Biksi I Szeredi L Gyurkovsky M. First record of autochthonous canine leishmaniasis in Hungary. Vector Borne Zoonotic Dis. (2012) 12:588–94. 10.1089/vbz.2011.0906 22607079 PMC3398396

[70] Percivalle E Cassaniti I Calzolari M Lelli D Baldanti F. Thirteen years of phleboviruses circulation in Lombardy, a Northern Italy Region. Viruses. (2021) 13:209. 10.3390/v13020209 33573092 PMC7911539

