# Correction: Monitoring of sandflies (Diptera: Psychodidae) and pathogen screening in Slovenia with habitat suitability modeling

**DOI:** 10.3389/fvets.2025.1696847

**Published:** 2025-10-29

**Authors:** Vladimir Ivović, Peter Glasnović, Sara Zupan, Tea Knapič, Tomi Trilar, Miša Korva, Nataša Knap, Urška Glinšek Biškup, Tatjana Avšič-Županc, Katja Adam

**Affiliations:** ^1^Faculty of Mathematics, Natural Sciences and Information Technologies, University of Primorska, Koper, Slovenia; ^2^Slovenian Museum of Natural History, Ljubljana, Slovenia; ^3^Institute of Microbiology and Immunology, Faculty of Medicine, University of Ljubljana, Ljubljana, Slovenia

**Keywords:** sandflies, monitoring, distribution, modeling, Slovenia

In the published article the **Funding** statement contained an error.

The number associated to CLIMOS Scientific Committee was displayed as “**CLIMOS026**.”

The correct statement reads:

The study was supported by the Slovenian Research Agency, Ministry of Health and Ministry of the Environment and Spatial Planning (grant no. V3-2313: Monitoring of vector borne pathogens in vectors in Slovenia and grant V3-1903: Establishment of monitoring of vectors and vector-borne diseases in Slovenia, P3-0083: Host-parasite relationship). This study is based on data processing funded by European Commission grant 101057690 and UKRI grants 10038150 and 10039289, while data collection was not covered by these grants. It is cataloged by the CLIMOS Scientific Committee, the number is CLIMOS027 (http://www.climos-project.eu). The funders had no role in study design, data collection and analysis, decision to publish, or preparation of the manuscript.

The original version of this article has been updated.

